# Polydimethylsiloxane as a Modifier of the Processing, Surface and Mechanical Properties of the Linear Low-Density Polyethylene Recyclate

**DOI:** 10.3390/ma18112552

**Published:** 2025-05-29

**Authors:** Arkadiusz Kloziński, Przemysław Postawa, Paulina Jakubowska, Milena Trzaskalska

**Affiliations:** 1 Institute of Chemical Technology and Engineering, Faculty of Chemical Technology, Poznan University of Technology, Berdychowo 4, 60965 Poznan, Poland; paulina.jakubowska@put.poznan.pl; 2 Department of Technology and Automation, Faculty of Mechanical Engineering, Częstochowa University of Technology, Armii Krajowej 21 St., 42201 Częstochowa, Poland; milena.trzaskalska@pcz.pl

**Keywords:** recycling, linear low-density polyethylene, polydimethylsiloxane, polymer recyclate, polymer blends, processing properties, surface properties, mechanicals properties

## Abstract

This study investigated the effect of adding polydimethylsiloxane (PDMS) on the processing, surface and mechanical properties of linear low-density polyethylene (rLLDPE) recyclate generated as post-production waste in the rotational molding process. Polymer blends containing 0.1, 0.2, 0.4, 1.0 and 2.0 wt.% of polydimethylsiloxane were produced during twin-screw extrusion, followed by cold granulation. The addition of the modifier at the adopted concentration range lowered the water absorption of the recyclate and contributed to a slight increase in processing shrinkage; however, it did not significantly affect its processability (MFR~const). The modification carried out increased the hydrophobic character of the recyclate surface (the wetting angle for water was enhanced) and decreased the value of the dynamic friction coefficient. It also contributed to an improvement in surface gloss. The deterioration of point hardness and scratch hardness of the recyclate was noted with an increase in the PDMS content in the mixture. The addition of polydimethylsiloxane caused changes in the nature of resulting cracks (increased width and reduced longitudinal deformation), which led to surface smoothing and increased the sliding effects. There was no negative effect of PDMS addition on the mechanical properties (static tensile) of the recyclate. The impact strength of rLLDPE deteriorated slightly. The research conducted shows the high application potential of PDMS as a modifier of the surface properties of low-density polyethylene linear recyclate and of selected processing properties, which can contribute to the shortening of the production cycle, thus potentially increasing its attractiveness compared to the original raw materials.

## 1. Introduction

The plastics industry undergoes constant, dynamic development in order to create improved materials and to meet the expectations of customers. The market is in need of products that are adapted to intended applications and exhibit very good properties. Currently, the development of new polymer materials is mainly based on changing the properties of existing polymers as a result of their chemical, physical or mixed modifications [1,2,3]. Presently, the thermoplastics sector is characterized by the highest global production rate, therefore modification of their properties has become one of the most important research challenges in the fields of polymer chemistry, polymer technology and materials science [4]. Changes in thermoplastic polymer properties can be achieved, e.g., during their processing, such as using reactive extrusion [5,6] and homogenization in the plasticized state (batch mixers, twin-screw extruders) [7,8]. The process of physical modification of polymers, which involves their homogenization in a plasticized state with various types of additives or other polymers, is widely used in the industry for the production of polymer blends [9] and polymer composites [10]. Polyethylene (PE) is one of the most commonly physically modified commercial thermoplastic polymers due to the wide spectrum of its properties and diversity of applications [11]. Additionally, this polymer is characterized by the highest global production, which amounted to approximately 110.13 million tons in 2022 [12]. Many varieties of polyethylene with different properties (i.e., low, medium or high densities as well as low, medium, high or very high molecular weights) can be physically modified using various types of admixtures [13]. These additives are very diverse in terms of their origin (natural, synthetic), chemical structure (organic and inorganic) and form (solid, liquid). However, the function and purpose of the used modifier and filler are the most important factors. Three main areas of change can be distinguished based on the purpose of polymer modification: changes in processing properties, structure and functional properties [13,14]. At the same time, these areas are very often involved in a direct cause-and-effect relationship; hence, changes in properties in one area are accompanied by a spontaneous change in properties in another area. An example of this phenomenon includes various types of fillers, the addition of which results in a strengthening effect in composites (improvement in functional properties), while also affecting their processing properties [15,16,17]. The various effects of the modifier additive on the polymer can also be revealed in one area of its properties [14]. Therefore, the search for and development of selective modifiers is a major research challenge.

The plastics industry is developing dynamically, creating better and better materials in order to meet the requirements of customers who expect raw materials that exhibit very good properties and are adapted to intended applications. Currently, the development of new polymer materials is mainly based on changing the properties of existing polymers as a result of their chemical, physical or mixed modification [1,2,3]. Currently, in the global polymer market, the largest production is in thermoplastics, so modifying their properties has become one of the most important research challenges in polymer chemistry, technology and materials science [4]. Among other things, changing the properties of thermoplastic polymers can be carried out during their processing, for example through reactive extrusion [5,6] and homogenization in the plasticized state (batch mixers, twin-screw extruders) [7,8]. The process of physical modification of polymers, involving their homogenization in a plasticized state with various types of additives or other polymers, is widely used in technologies for the production of polymer blends [9] and polymer composites [10]. One of the most commonly physically modified commercial thermoplastic polymers is polyethylene (PE). This is due to the wide spectrum of its properties, diversity of applications [11], and above all, the fact that it is the polymer with the largest global production, which in 2022 amounted to approximately 110.13 million tons [12]. Many varieties of polyethylene with different properties, low, medium or high densities, low, medium, high or very high molecular weights, can be physically modified using various types of admixtures [13]. The additives used are very diverse in terms of their origin (natural, synthetic), chemical origin (organic and inorganic) and form (solid, liquid). However, the most important is the function and purpose of the modifier and filler used. From the point of view of the purpose of polymer modification, three main areas of change can be distinguished: change in processing properties, structure and functional properties [13,14]. At the same time, these areas are very often in a direct cause-and-effect relationship, and changes in properties in one area are accompanied by a spontaneous change in properties in another. An example of this can be the use of various types of fillers, whose addition produces a strengthening effect in composites (improvement in functional properties), while affecting their processing properties [15,16,17]. The various effects of the modifier additive on the polymer can also be revealed in one area of its properties [14]. Therefore, a major research challenge is the search for and development of selective modifiers.

Organosilicone polymer compounds, which effectively modify the surface properties of polyolefins, include polydimethylsiloxane (PDMS) [18,19,20]. PDMS is a polymer of great interest in various fields due to its outstanding advantages, such as non-toxicity, environmental friendliness [21], biocompatibility [21,22,23,24,25], hydrophobicity [20,21,26,27], chemical and thermal resistance [28,29,30], UV resistance [31], flexibility [21,27,28,32], low cost [33] and good mouldability [26,28,33]. As a result of the above-mentioned properties, PDMS is the most widely used silicone based on organic polymers, which exhibits the characteristics of a transparent, flexible compound with biocompatible properties [34,35]. It is used, e.g., in the production of lubricants [36], coatings [37], membranes [38,39], optical materials [40,41], microtransmitters [42], dressings [43], implants [25] and other products [22,26,35]. The excellent hydrophobic properties of PDMS (surface tension of approx. 20 Nm/m) are associated with the presence of methyl (-CH_3_) groups in its structure [35]. The anti-adhesive properties of PDMS are used in various polyolefin processing techniques, where it can be applied as both an internal and external release agent [44]. The addition of PDMS to polyolefins processed in the extrusion process reduces melt flow instability, improves the quality of the extrudate and reduces the energy input of the process [19,45]. Polydimethylsiloxanes with lower molecular weight (lower viscosity) are also widely used in the processing of plastics as external release agents on the mold surface (injection molding, rotational molding), facilitating the removal of the product [44,46]. However, it should be highlighted that the application of external release agents involves an additional technological process (the use of spray equipment—manual or automatic), which increases the duration of the production process. Internal release agents eliminate the mold preparation time necessary for applying the external agent and the time needed to apply the agent [44]. Therefore, there is ongoing research into effective internal release agents in polyolefin processing, including rotational molding, which would allow to speed up the technological process, increase production efficiency, reduce labor and decrease the energy input. Doping with PDMS allows the achievement of these specific goals during the modification of polyethylene, including linear low-density polyethylene (PE-LLD). The use of PDMS as an internal release agent facilitates the removal of products from molds during the rotational molding process, which leads to faster product manufacturing and reduced labor intensity—improving the economics of the process [18].

Most of the scientific reports which analyzed the effect of the PDMS additive on the surface properties of polyolefins focused on the modification of pure polymers [18,19,20,25]. Numerous inquiries from the industrial sector regarding attempts to modify/refine recyclates from the rotational molding process with PDMS as an additive, combined with a lack of publications describing changes in surface properties of polyolefin recyclates as a result of doping with PDMS, were the reason for undertaking this research within the framework of this study. Demonstrating the positive effect of PDMS addition on the above-mentioned properties of secondary polymer materials may contribute to their increased competitiveness relative to the original polymers. This type of research can be a source of theoretical insight in the area of recycling polymeric materials, as well as a source of practical knowledge on how polymeric recyclates can potentially be modified/refined to increase their marketability relative to commercial original pellets. Improvement in the functional properties of a recyclate can potentially increase its share in subsequent product life cycles, which is consistent with the concept of circular technology—aiming to increase recycling rates and improve the quality of recyclates [47,48]. Therefore, this study will discuss the effect of the PDMS additive on the processing, surface and mechanical properties of linear low-density polyethylene recyclate.

## 2. Materials and Methods

### 2.1. Tested Materials

A recyclate of linear low-density polyethylene (rLLDPE) in the form of cylindrical regular pellets, produced by twin-screw extrusion technology, was used for the research. The material originated from post-production waste (primary recyclate) obtained from the rotational molding process (process rejects, offcuts and elements remaining after the tanks have been exposed). The material was characterized by a distinguishing recyclate odor, black color (the color of waste batches), a density of 0.944 g/cm^3^ (23 °C) and a melt flow rate (*MFR*_2.16, 190_) of 4.04 g/10 min (own research). The recycling process was carried out at iPP Sp. z o.o. (Koscielec, Poland).

The polyethylene was doped with ultra-high molecular weight (UHMW) polydimethylsiloxane (PDMS). A commercial product in the form of a concentrate (50% wt. PDMS) on an low-density polyethylene (LDPE) carrier (LDPE/PDMS), manufactured by Dupont (Wilmington, DE, USA) under the trade name MULTIBASE^TM^ MB50-002 (Silicone Masterbatch), was used in the studies. The concentrate was characterized by a density of 0.977 g/cm^3^ (23 °C) and a melt flow rate (*MFR*_2.16, 190_) of 12.77 g/10 min (own research).

### 2.2. Sample Preparation

The physical modification of the polyethylene recyclate with PDMS was conducted during the homogenization process in a cold granulation extrusion technological line. A twin-screw extruder, model ZM/258/21 by Zamak Mercator (Skawina, Poland), with a screw diameter of 20 mm (d = 20 mm) and an l/d ratio of 40 was used. An rLLDPE regranulate was produced, which contained 0.1, 0.2, 0.4, 1.0 and 2.0 wt.% of the PDMS. The precise compositions of the modified polyethylene recyclate samples and their denotations, which will be used later in the text, are shown in Table 1.

Specimens with dimensions according to ISO 527, ISO 179-1 and ISO 294-1 were formed using a Battenfeld PLUS 35 (Vienna, Austria) hydraulic injection molding machine operating at the maximum processing temperature of 190 °C.

### 2.3. Analytical Methods

The moisture content of the materials was determined by the gravimetric method. The dried polymer regranulate was placed in an unheated warehouse in the technological hall (average daily temperature of 5.6 °C, relative humidity of 83%) for 76 h. Then, samples (approx. 5 g) were dried for 3 h at 100 °C using a laboratory dryer with hot air circulation, Memmert, model SF 75 (Schwabach, Germany). The moisture content was determined based on the difference in the mass of the polymer samples before and after drying. The mass of the samples was determined using an analytical weight, Axis, model AD200 (Gdansk, Poland).

The effect of the PDMS addition on the mass flow rate (*MFR*) index, which constitutes a technical measure of the processability of thermoplastic polymers, was also determined. The measurements were conducted using a Dynisco plastometer, model LMI 4004 (Franklin, TN, USA), according to the applicable ISO 1133 standard. The measurement temperature was equal to 190 °C, and the piston loading was 2.16 kg. The *MFR* value was determined from the equation below [14]:

(1) $${MFR}_{\left(\Theta ,\;M_{nom}\right)}=\frac{t_{ref}\cdot m}{t}\;(g/10\;\min)$$

where Θ—measurements temperature, $M_{nom}$—nominal piston load, *t_ref_*—reference time (10 min), t—time after which the extruded polymeric material stream should be cut off, *m*—average mass of extruded polymeric material sections.

Rheological analysis was performed using an Anton Paar MCR 301 rotational rheometer (Graz, Austria). The tests were conducted in rotation mode, for a cone-plate measuring system with a diameter of 25 mm and an angle of 2°, with a gap width of 103 μm. The rheological characterization of the test materials (the viscosity curve $\eta =f(\dot{\gamma })$ [14]) was carried out at a shear rate ($\dot{\gamma }$) ranging from 0.001 to 100 s^−1^.

Samples for the evaluation of processing shrinkage of molded parts were produced using an experimental injection mold with replaceable inserts, compliant with ISO 294-1. A detailed description of the mold is presented in publications [49,50]. The molded parts were characterized by the following dimensions: 60 mm × 60 mm × 2 mm. A stand for sample dimension measurement was equipped with a VIS SYLVAC SYSTEM 0–25 mm electronic digital sensor with an accuracy of 0.001 mm (Yverdon, Switzerland). Before the measurement, the station was calibrated to a dimension of 60 mm using two gauge blocks of 10 and 50 mm. The tests determined the longitudinal shrinkage (*SL*) after 16 h and 3 months after the end of the injection process.

The measurements of the wetting angle were performed at a temperature of 23 ± 2 °C and air humidity of 30 ± 2%. The volume of the water droplet used for measurement was 4 µL. The wetting angle was measured immediately following the application of a droplet of the measuring liquid on the sample surface (after a few seconds). The measurements were conducted using an OCA15EC goniometer manufactured by DataPhysics Instruments GmbH (Filderstadt, Germany) and a piece of software for computer image analysis. The analysis was performed on 5 samples for each material type.

The determination of dynamic coefficient of friction (*µ_D_*) [51] was performed according to the ISO 8295 testing standard. A universal testing machine, ZwickRoell Z020TH AllroundLine (ZwickRoell GmbH & Co. KG, Ulm, Germany), with a 100 N tensile strength measurement head and a special sample holder for the friction coefficient determination test, was applied for the mechanical tests. The tensile rate (the speed of friction causing motion) was 100 mm/min. The moldings, with dimensions of 60 mm × 60 mm × 2 mm (attached to a measuring trolley), were moved across a polyethylene film with a dynamic coefficient of friction of 0.14.

Fourier Transform Infrared Spectroscopy (FTIR-ATR) was used to identify the presence of PDMS on the surface of the prepared polymer blends. Infrared spectra were collected using a Nexus Nicolet 5700 Fourier Transform Infrared Spectrophotometer (FTIR, Thermo Electron Scientific Instruments Corporation, Madison, WI, USA) equipped with an attenuated total reflection (ATR) accessory with a diamond crystal, and measurements were taken at room temperature in the range of 4000–525 cm^−1^, with a resolution of 4 cm^−1^ and 32 scans.

Energy dispersive X-ray spectroscopy (EDS) was used to confirm the presence of PDMS on the surface of the produced polymer blends. The maps of silicon (Si) elements were performed using the Hitachi HT7700 (Hitachi, Tokyo, Japan) operating in STEM mode and equipped with an energy-dispersive X-ray microanalysis system (Thermo Scientific, Waltham, MA, USA).

The hardness of rLLDPE and its modified compositions was evaluated using a static Shore hardness tester from Zwick (ZwickRoell GmbH & Co. KG, Ulm, Germany), according to the ISO 868 testing standard.

The scratch hardness tester Lineartester (model 249) from Erichsen was used to assess the scratch resistance of the recyclates. All tests were carried out in accordance with ISO 1518-1 using a 1 mm diameter stylus, moving at a speed of 35 mm/s and a load of 14 N. The measurements of the depth of penetration during scratch tests of the manufactured samples were conducted using a digital microscope VHX-900F from Keyence (Osaka, Japan). The microscope was equipped with a motorized Z axis and a digital image processing processor, which allowed for achieving a very high depth of focus while simultaneously measuring the surface profile. A lens with a magnification range of 100–1000 times equipped with a fiber optic illuminator was used during the observations. On the basis of the measured scratch width and load (14 N), the scratch hardness was calculated using the following equation [52,53]:

(2) $$H=\frac{4\cdot x\cdot L}{\pi \cdot {\left(w\right)}^{2}}$$

where

- x is the parameter specifying the nature of the tip contact with the coating (1 < x < 2 for viscoelastic or viscoplastic deformation, x = 1 for purely elastic contact and x = 2 for plastic deformation);
- L represents the tip load, and w is the scratch width. In this study, the x parameter was taken as 1.

The effect of PDMS addition on the gloss of rLLDPE was determined using a glossmeter from TestAn, series DT-268 (Gdansk, Poland). The measurement was carried out in accordance with the ASTM D2457 standard, using a measurement angle of 60°.

Color measurement was performed using a Precision Colorimeter, model NR 145 (Guangdong ThreeNH Technology Co., Ltd., Shenzhen, China), with a measurement geometry of 45°/0°. The CIE Lab color space was used for color analysis.

Mechanical properties under static elongation conditions, such as tensile modulus (*E_t_*), tensile strength (*σ_M_*) and elongation at break *ε_B_* were evaluated for the samples by means of the static tensile test, according to the ISO 527 standard. The tensile tests were performed using a ZwickRoell Z020TH AllroundLine universal testing machine. The traverse speed was set to 1 mm/min during the determination of the *E_t_* and 100 mm/min during the remaining part of the test.

Charpy impact strength tests were performed on notched specimens using the CEAST/Instron Charpy impact testing machine, model 9500. The adopted test method was carried out according to the ISO 179 standard. Impact loading was carried out with a 5.0 J pendulum.

## 3. Results and Discussion

### 3.1. Processing and Rheological Properties

From an application perspective, it is essential to know how the use of a specific additive will influence the properties of the polymer (either favorably or adversely) and whether any changes other than those expected will occur. In this respect, processing properties and processing preparation processes, such as drying, are a priority. Therefore, the investigation also involved the determination of the effect of PDMS addition on the basic parameters associated with rLLDPE processing, such as moisture absorptivity, melt flow ratio, viscosity and processing contraction. The obtained results are summarized in Table 2. Exposure of the test materials to weather conditions characteristic for unheated storage rooms in the autumn season (in Central and Eastern Europe) showed a positive influence of recyclate modification on the reduction of its moisture absorptivity. The recommended minimum moisture content of polyethylene, prior to its processing, falls within the range of 0.03 to 0.05 wt.% [54]. As shown by the figures in Table 2, the moisture content decreased with increasing amounts of PDMS in the polymer mixture. This effect is already noticeable at a PDMS addition of 0.1 wt.%. At the highest adopted modifier concentrations, the moisture content is below 0.5 wt.% (0.045 wt.% for 1.0 PDMS and 0.017 wt.% for 2.0 PDMS), which is the permissible value for processing without additional drying. The hydrophobic properties of the applied modifier [55] allow for a reduction in drying time for all modified recyclates prior to their processing and even eliminate the necessity for drying in case of systems containing 1.0 and 2.0 wt.% PDMS. Observations of variations in moisture absorptivity showed that the addition of PDMS to the LLDPE recyclate may contribute to a decrease in energy expenditures during the preparation processes by reducing them, thereby speeding up the technological process and resulting in economic benefits.

**Figure 1:**
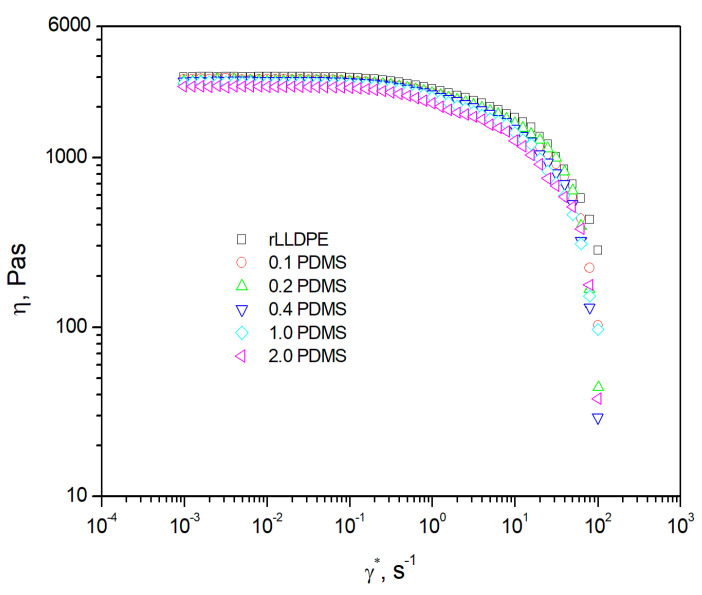
Viscosity curve for rLLDPE and rLLDPE modified with PDMS (0.1 PDMS–2.0 PDMS).

The evaluation of the effect of PDMS addition on the processability of rLLDPE was carried out by measuring the low melt flow rate, which is a technical measure of the processability of thermoplastic polymers [56]. The determined *MFR* values (Table 2) for individual systems containing PDMS in the considered range of concentrations did not show any significant influence of the modifier on the analyzed property. The noted variations were contained within the standard deviation range. The melt flow rate for unmodified polyethylene was 4.04 ± 0.033 g/10 min. For a mixture containing 2 wt.% PDMS (2.0 PDMS), the value of *MFR* remained at a level of 3.98 ± 0.036 g/10 min. The gradual addition of an increasing amount of PDMS did not result in a steady increase in the *MFR* of the mixture, which could be expected considering the *MFR* value of the modifying concentrate (12.77 g/10 min). The absence of any significant changes in the *MFR* value for mixtures with a PDMS addition suggests that the addition of the modifier in the adopted range of its concentrations does not significantly affect the processability of polyethylene recyclate. The values of the melt flow rate of the recyclate and mixtures containing PDMS are within the range of 2 to 10 g/10 min, which means that they exhibit a processability comparable to mixtures used in the rotational molding process [57,58]. Thus, they can be used as a raw material for the aforementioned processing. It should be underlined that the conditions prevailing under plastometer flow conditions may differ from those occurring in technological processing, and the obtained measurement results can deviate from the behavior of polymers under processing conditions [56]. Using a measurement methodology which enables the determination of the shear rate for the flow of material in the plastometer channel [14,56], it was possible to determine the value of $\dot{\gamma }$ occurring during the flow of the analyzed compounds in the conducted measurements. According to information provided in the literature, melt spinning processes are accompanied by flows with very low shear rate values, which do not exceed 20 s^−1^ [57,58]. For rLLDPE and PDMS-containing mixtures, shear rate during the flow through the cylindrical die of the plastometer was equal to approx. 9 s^−1^. Therefore, it can be inferred that the *MFR* values determined under the adopted measurement conditions (piston load 2.16 kg, temperature 190 °C) will reflect (to a considerable extent) the behavior of the test materials in the technological conditions of the melt spinning process, and that the modification should not affect the processing behavior. As is known, the flow rate index is a measure of the fluidity of a polymer, which is the reciprocal of its viscosity, and it represents a single point on the material’s viscosity curve determined at the measurement temperature [59]. From a practical point of view, the polymer’s zero viscosity value plays a significant role in melt spinning processes. It is recommended that LLDPE used in melt spinning be characterized by a low zero viscosity value, which allows the development of a product with satisfactory surface quality, free from the so-called ‘pin-hole’ effect occurring as a result of retaining air bubbles between sintering polymer particles [58,60]. Therefore, in order to examine the influence of the performed modification on the viscosity of rLLDPE, the viscosity curves of the test materials were determined at low shear rates ranging between 0.001 and 20 s^−1^, and at a temperature of 190 °C. The obtained viscosity curves are juxtaposed in Figure 1. In the tested shear rate range, viscosity curve shapes characteristic of non-Newtonian pseudo-plastic liquids were obtained [59,61,62]. The phenomenon of shear thinning starts for all materials at the $\dot{\gamma }$ value of approx. 0.1 s^−1^. A slight shift in the curves $\eta =f(\dot{\gamma })$ toward lower viscosity values is observed with increasing PDMS content in the polyethylene recyclate. This is confirmed by the zero viscosity figures summarized in Table 2. The unmodified recyclate was characterized by a zero viscosity of 3010 Pa·s. The influence on the interaction of polyethylene macroparticles, which resulted in a reduction in internal flow resistance and, as a consequence, a decrease in rLLDPE viscosity, is already observed at a PDMS addition of 0.1 wt.%. The addition of the lowest adopted modifier amount caused a reduction in the zero viscosity of the recyclate by 80 Pa·s. For the recyclate containing the highest amount of PDMS, the zero viscosity was lowered to a level of 2640 Pa·s. It can be presumed that the reduction in viscosity is a result of polyethylene–polydimethylsiloxane interactions similar to those occurring with the addition of plasticizers [63] or slip agents [14]. Similar to a plasticizer, PDMS penetrates into the spaces between the rLLDPE chains, contributing to a reduction in the force of intra- and intermolecular interactions between them. This, in turn, results in a reduction in internal flow resistance, which in practice is reflected by a decrease in viscosity. The reduction in viscosity as a result of the performed modification may give rise to an improvement in the surface quality of products obtained in melt spinning process by favoring the process of recyclate granules and reducing the number of imperfections occurring as a consequence of entrapping air bubbles in the structure of the product’s wall.

The processing shrinkage is an important parameter that should be considered in processing, and primarily in the design of processing tools, including molds. In the course of the investigation, the longitudinal processing shrinkage (*S_L_*) was determined after, respectively, 16 h and 3 months from the fabrication of standardized samples during the injection molding process. The *S_L_* values summarized in Table 2 show that the longitudinal processing shrinkage of unmodified polyethylene recyclate is contained in the range of contraction characteristic of original linear low-density polyethylenes (2.0~2.5%) [64], amounting to 2.11 ± 0.117% (after 16 h) and 2.22 ± 0.105% (after 3 months). The modification of the polymer causes a slight increase in longitudinal (in flow direct) shrinkage, proportional to the increase in the PDMS content in the recyclate. For the mixture with the highest modifier content (2.0 PDMS), the *S_L_* value increases by approx. 0.4%, amounting to 2.51 ± 0.177% (after 16 h) and 2.61 ± 0.180% (after 3 months), respectively. The higher values of secondary shrinkage (after 3 month) for mixtures with 2% of PDMS show that the modifier facilitates the long-term relaxation of residual stresses during polymer molding. The slight shrinkage increase can be a consequence of the presence of low-density polyethylene in the mixture, which is characterized by a higher shrinkage value (2.0~4.0%) [64] compared to linear low-density polyethylene. As shown by the shrinkage measurements, the increase in the *S_L_* value represents an unfavorable effect of the performed rLLDPE modification (material effect), and it should be taken into consideration during the fabrication processes in order to ensure that products characterized by satisfactory dimensions are obtained.

### 3.2. Surface Properties

**Figure 2:**
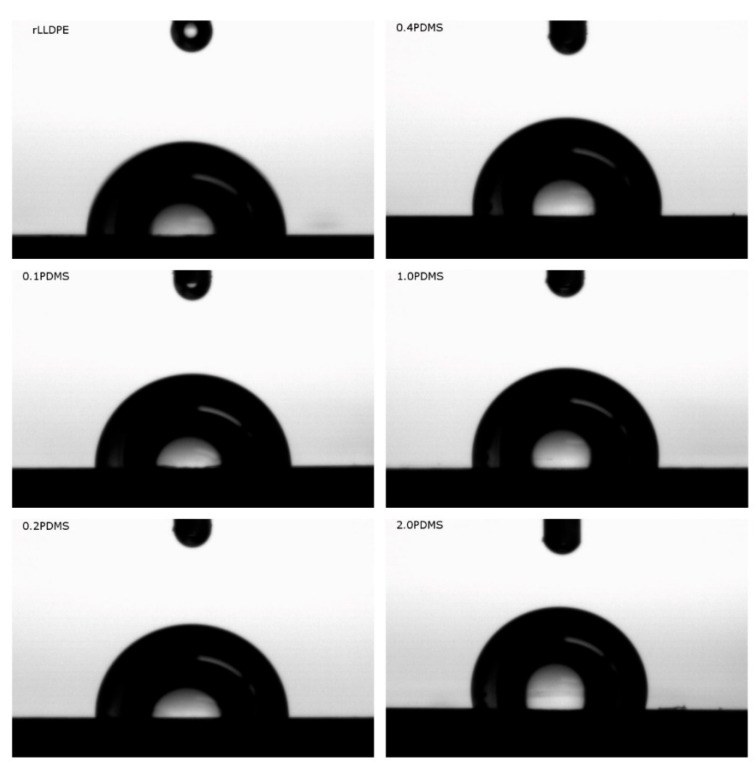
Sample images of water drops deposited on the surface of research materials.

The main objective of the modification of the linear low-density polyethylene recyclate with the addition of PDMS was to reduce the wettability of the polymer’s surface in order to facilitate the removal of large-size rotational products of the molding process from the mold. Due to the presence of recurring non-polar -CH_2_- fragments in its structure, polyethylene is classified as a hydrophobic material [13], which means that it is poorly wettable and theoretically should not pose any major problems during the removal of products from the mold during processing. However, for recyclates, the de-molding of products may cause more difficulties compared to newly synthesized compounds due to changes in properties resulting from the presence of impurities [65] or caused by the degradation of the polymer [66]. Therefore, attempts to modify the recyclate with the aim of increasing it hydrophobicity seem justified, as this may speed up its processing compared to the original polyethylene. Changes in the surface hydrophobicity of rLLDPE were determined based on the wettability angle, the values of which are summarized in Table 3. The recyclate alone exhibits a water contact angle that is characteristic of commercial linear low-density polyethylenes [67,68], i.e., in the range of 88~94°. Example images of water drops deposited on the surface of the research materials are presented in Figure 2. A change in polyethylene surface wettability is already observed at a PDMS content of 0.1 wt.% (with the wettability angle increasing to 90.27 ± 0.767° from 88.25 ± 0.257°), which confirms the high effectiveness of the applied modification, and the observations are consistent with the results of studies carried out using original polymers [18,19]. The wettability angle increases with increasing PDMS content in the mixture, reaching the highest value, i.e., 102.47 ± 0.753°, for 2.0 PDMS. The increase in the hydrophobic character of the polyethylene recyclate surface is accompanied by a decrease in the dynamic friction coefficient (the second row in Table 3), which is practically associated with a reduction in friction force between plates made of the modified polymer and standard polyethylene film. In the adopted modifier concentration range (from 0.1 to 2.0 wt.%), the value of the dynamic friction coefficient varies within the range of 0.13 ± 0.011 to 0.06 ± 0.004. The addition of 2.0 wt.% PDMS reduced the dynamic friction between the examined surfaces by more than 50%. By juxtaposing the above-mentioned test results for the variations in surface hydrophobicity and the dynamic friction coefficient of rLLDPE with the relationships provided by the authors of [18], it can be inferred that the addition of PDMS will increase the efficiency of product removal from the mold and contribute to an acceleration of rotational molding process.

**Figure 3:**
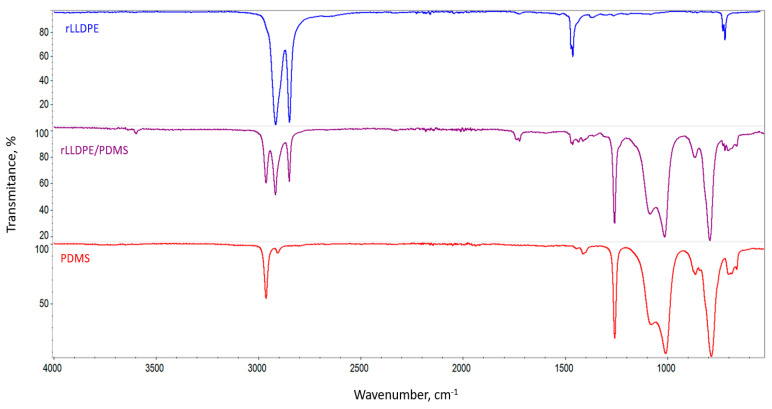
FTIR-ATR spectra of rLLDPE, PDMS and rLLDPE/PDMS concentrate containing 50 wt.% PDMS.

The above-mentioned changes in the recyclate’s hydrophobicity (increase in the wettability angle) and dynamic coefficient of friction (decrease in µD↓) are a consequence of an increased presence of PDMS on the surface of rLLDPE, along with an increase in the modifier content of the produced blends—which is confirmed by the FTIR spectra and EDS maps. PDMS, the PDMS/LDPE concentrate and rLLDPE were characterized using ATR-FTIR in order to evaluate and compare the chemical groups in polydimethylsiloxane and recycled low-density polyethylene (Figure 3).

**Figure 4:**
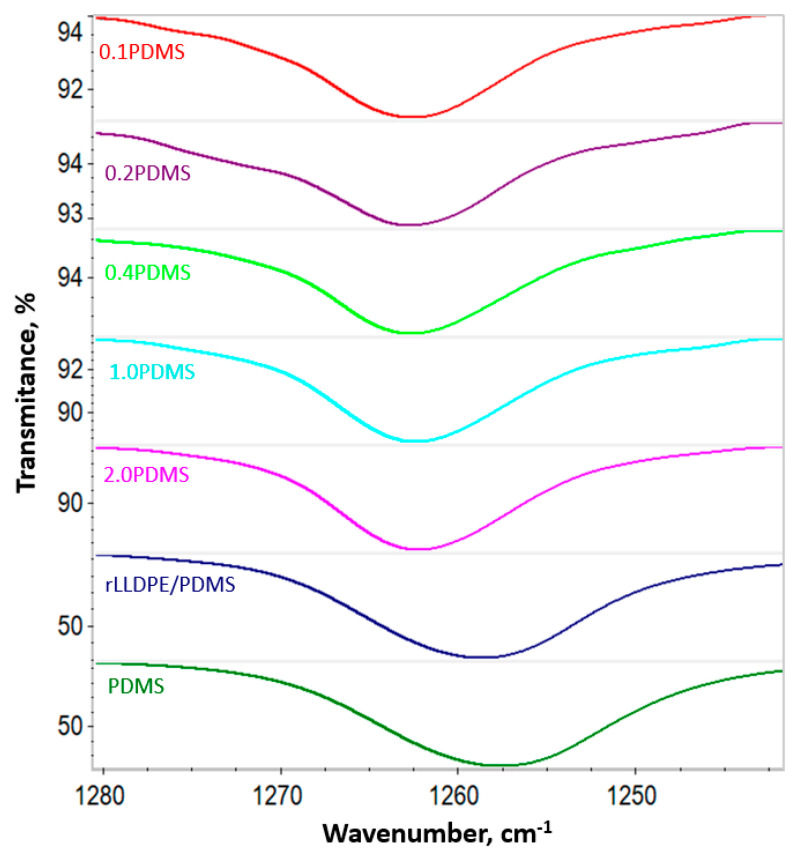
FTIR-ATR spectra in the wavenumber range of 1280–1240 cm^−1^, i.e., in the range of the Si-CH_3_ bond absorption band for pure PDMS, rLLDPE/PDMS concentrate and the tested polymer blends containing different concentrations of PDMS (0.1, 0.2, 0.4, 1.0 and 2.0 wt.%).

PDMS exhibited IR peaks at 789–796 cm^−1^ (−CH_3_ rocking and Si-C stretching in Si-CH_3_), 1020–1074 cm^−1^ (Si-O-Si stretching), 1280–1259 cm^−1^ (CH_3_ deformation in Si-CH_3_) and 2950–2960 cm^−1^ (asymmetric CH_3_ stretching in Si-CH_3_) [69,70,71]. The spectrum for rLLDPE shows IR peaks at 717 cm^−1^ (CH rocking in –CH_2_–), 1377 and 1470 cm^−1^ (symmetric and asymmetric CH bending), 2845 and 2915 cm^−1^ (symmetric and asymmetric –CH_2_– stretching) [72,73]. The spectrum for the rLLDPE/PDMS concentrate consists of characteristics peaks for both the above-mentioned materials (PDMS and rLLDPE). The FTIR-ATR spectra presented in Figure 4 are in the wavenumber range of 1280–1240 cm^−1^, i.e., in the range of the Si-CH_3_ bond absorption band for pure PDMS, rLLDPE/PDMS concentrate and the tested mixtures containing different concentrations of PDMS (0.1; 0.2; 0.4; 1.0; 2.0 wt.%). Shifts in the peak position with increasing PDMS concentration in the polymer matrix indicate an increase in the amount of modifier on the rLLDPE surface, which is the result of an enhanced interaction between Si-CH_3_ groups [74].

**Figure 5:**
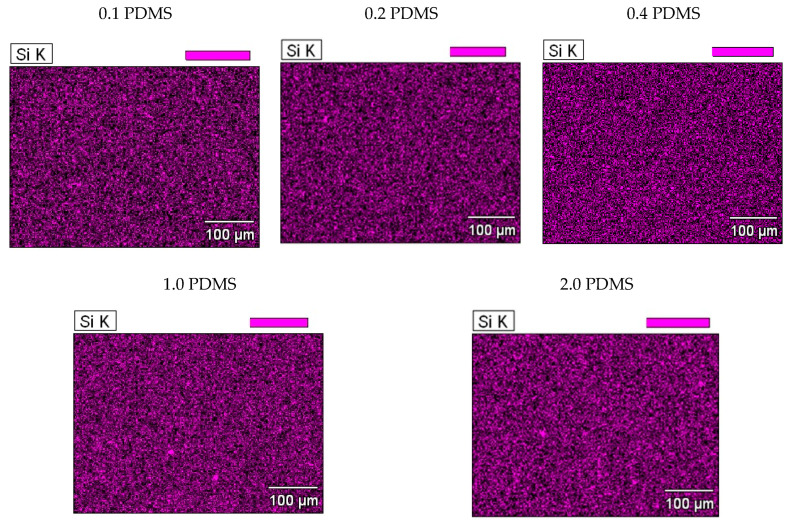
EDS mapping of the tested polymer blends containing different concentrations of PDMS (0.1, 0.2, 0.4, 1.0 and 2.0 wt.%).

The presence of PDMS on the surface of the polymer blends was also confirmed by energy dispersive X-ray spectroscopy (EDS). EDS analysis of the polymer blends surface identified the element silicon (Si), which is consistent with the composition of PDMS (Figure 5). The image of the obtained maps shows the uniform distribution of silicon on the surface of blends containing 0.1, 0.2 and 0.4 wt.% PDMS. In mixtures containing the highest adopted concentrations of the modifier, i.e., 1.0 and 2.0 wt.%, the formation of a few small Si clusters is observed on their surface. This indicates a decrease in the homogeneity of the blends and a deterioration in the degree of homogenization of PDMS in the polyethylene matrix and on the surface of the moldings, for the above-mentioned PDMS concentrations. However, this did not translate into a measurable effect on the surface properties of the mixtures; it did not cause an increase in standard deviations in the values of the determined parameters, e.g., wettability angle or dynamic coefficient of friction (see Table 3).

**Figure 6:**
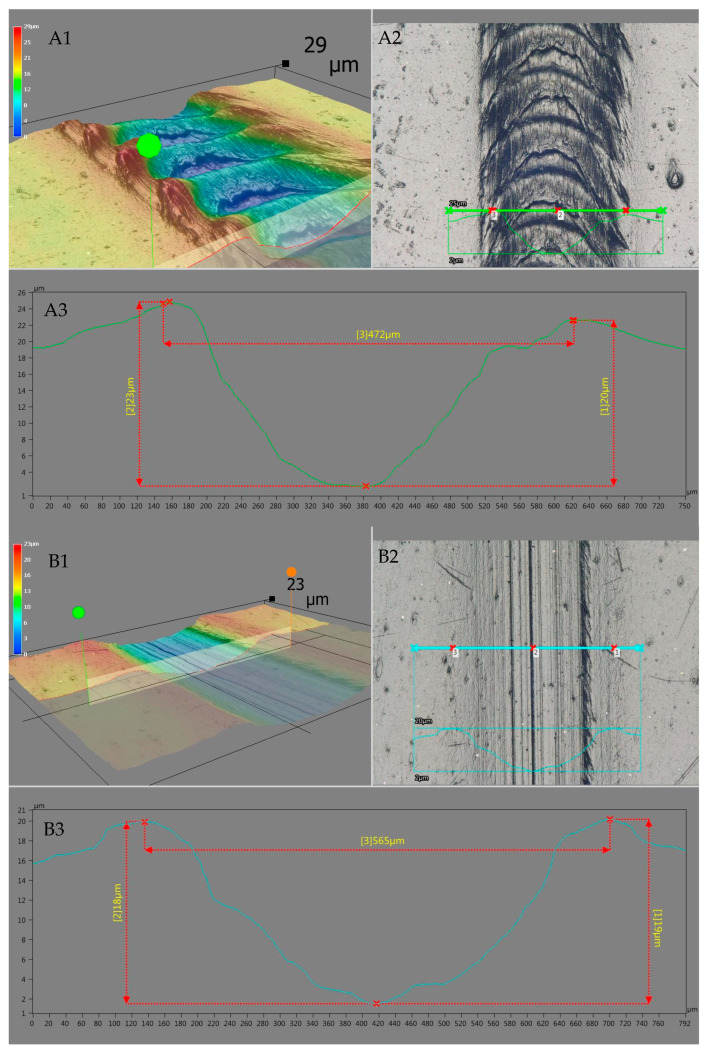
Microscopic analysis images of scratches for rLLDPE (**A1**–**A3**) and modified rLLDPE with PDMS (2.0 PDMS) (**B1**–**B3**)—scratch width analysis; (**A1**,**B1**)—digital spatial representation with depth attribute; (**A2**,**B2**)—real scratch image from the microscope; (**A3**,**B3**)—dimensioned profile of the indicated cross-section.

From the perspective of the usefulness of products made of plastics, an important role is played by their resistance to mechanical factors that may impair the aesthetic qualities of their surface, e.g., as a result of spot deformation or scratching. Therefore, the investigation also included the determining the influence of the employed modification of polyethylene recyclate on the surface hardness of the molded pieces. Within the measurements, the Shore hardness and scratch hardness of rLLDPE and the produced mixtures were determined, and the obtained results are summarized in Table 3. As demonstrated, the performed modification does not affect the Shore hardness of polyethylene up to a PDMS content of 0.4 wt.%. In this concentration range, variations in hardness are within the margin of measuring error. A slight decrease in hardness (by approx. 1°) is observed at a PDMS content of 1.0 wt.% and by 2° for a mixture containing 2.0 wt.% modifier. The resistance to spot damage is not impaired significantly. A different situation is observed for scratch hardness, which reflects damage occurring on the surface due to scratching. A reduction in the scratch hardness of polyethylene already takes place at a modifier addition of 0.1 wt.%. For unmodified polyethylene, scratch resistance is at 79.8 ± 0.39 MPa, while for a compound with 0.1 PDMS, it lowers to a value of 63.2 ± 0.24 MPa. The scratch resistance of rLLDPE drops with the increase in PDMS content, reaching its lowest recorded value, i.e., 55.7 ± 0.20 MPa, for a system containing 2 wt.% of the modifier. On account of the adopted measurement methodology, the obtained scratch hardness value does not reflect the first noted surface damage (scratching) [52], but it shows changes occurring in surface scratching under a constant load of 14N. With the increase in the PDMS content, the polyethylene surface became increasingly scratched, as illustrated by microscopic images. Example pictures of surface scratches, along with the determined profiles of changes which occurred due to scratching, specimens of unmodified linear low-density polyethylene (rLLDPE) and of recyclate containing 2.0 wt.% modifier (2.0 PDMS), respectively, are presented in Figure 6. The images of microscopic scratch analysis show the digital spatial mapping with a depth attribute (upper left part), an actual microscopic picture of scratching (on the right) and the dimensioned profile of the indicated cross-section (below).

**Figure 7:**
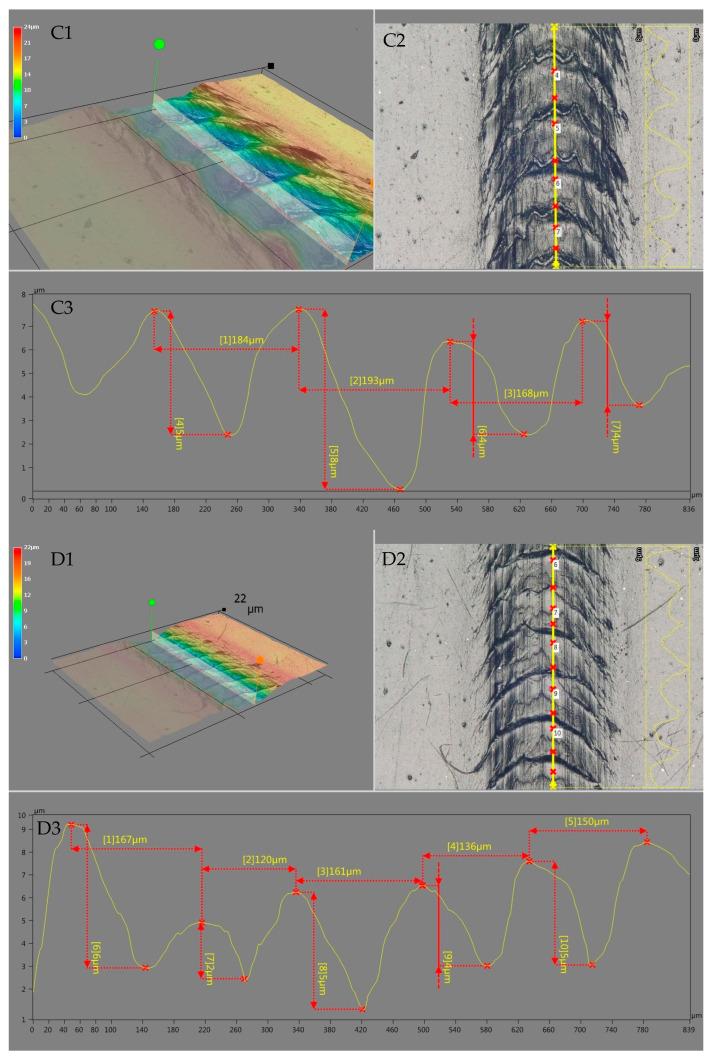

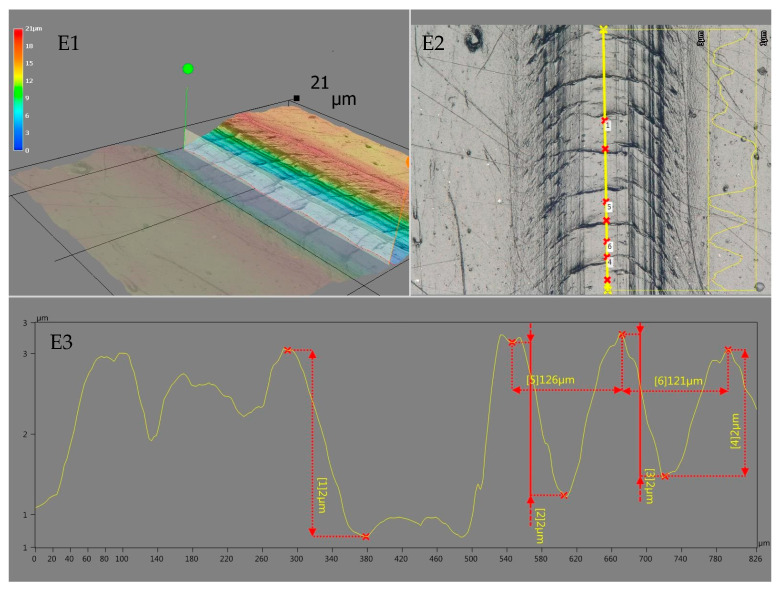
Microscopic analysis images of scratches for rLLDPE (**C1**–**C3**) and modified rLLDPE with PDMS (0.4 PDMS) (**D1**–**D3**) and (1.0 PDMS). (**E1**–**E3**)—scratch width analysis; (**C1**–**E1**)—digital spatial representation with depth attribute; (**C2**–**E2**)—real scratch image from the microscope; (**C3**–**E3**)—dimensioned profile of the indicated longitudinal section.

The analysis of the scratches that occurred showed variations in their width with the increase in the PDMS content in the polyethylene recyclate, which is reflected by the scratch hardness drops presented in Table 3. For unmodified polyethylene, acting with a scriber under a load of 14 N resulted in a scratch characterized by a width of approx. 472 μm (A3), and the scratch width increased up to approx. 565 μm (B3) for a mixture containing 2 wt.% modifier, which translates to an increase in its lateral dimension by approx. 20%. The width of the scratches increased proportionally to the modifier content. The use of the imaging technique with the capability of digital spatial mapping with a depth attribute allowed demonstration of a variation in the character of the scratches that occurred. The addition of PDMS increased the width of the scratch and also influenced its depth and the structure of the deformations that occurred (see A1 and B1) in the direction of scratch propagation (scratch axis). Increasing the modifier content in the polymer resulted in a reduction of polyethylene plastic deformation effects (undulations/distortions) caused by the movement of the needle during scratch testing. An analysis of the image in the scratch axis allowed for a better imaging of the variations in the deformations that occurred. Example results of this analysis are shown in Figure 7.

As indicated by the data regarding the dimensional profile of the deformations that occurred (deformation height, distance between deformation vertices), the most extensive deformations occurred along the axis of the scriber movement along the surface of the unmodified polyethylene recyclate. In the case of rLLDPE (C3), the deformations were characterized by an average height of approx. 5.3 μm, while the mean value of the distance between vertices was approx. 182 μm. The addition of 0.4 wt.% PDMS (D3) reduced the deformations to a level of approx. 4.4 μm (mean deformation height) and 147 μm (mean distance between deformation vertices). The parameters were further reduced in the case of a mixture containing 1.0 wt.% (E3), amounting to 2.0 μm and 123.8 μm, respectively. After comparing the values of the obtained scratch hardness (Table 3) and changes in the character of occurring scratches, it can be established that the addition of the employed modifier impairs the scratch hardness of polyethylene recyclate, causing the formation of scratches characterized by a larger width. At the same time, this contributes to a reduction in deformations/distortions occurring along the scratching axis. Thus, it improves the sliding properties of the polymer, which was actually confirmed by the results of the dynamic friction coefficient tests discussed earlier. This feature is important and desirable for materials which are expected to exhibit adequate scratch resistance in applications.

Polymers are usually transparent or white. To achieve the intended visual, marketing or decorative effect, a specific color is given to them. Polymers acquired after recycling processes are very often dyed black, as this color efficiently equalizes different parts of the material, masking their previous variations in color. The commercial recyclate of linear low-density polyethylene employed in this investigation was a black-colored material. The PDMS concentrate on the LDPE support used for modification was white. The addition of PDMS in the adopted concentration ranges did not cause any significant color changes, as determined based on the CIE Lab mathematical model. The change in the ∆*L* parameter, or brightness (luminance), for the highest modifier addition (2.0 PDMS) amounted to 0.6. The most notable difference between color hues, or the value of the ∆*E*, parameter occurred between rLLDPE and the surface of a mixture containing 2 wt.% PDMS, assuming a value of 0.76. In line with the relevant literature, when the differences between color hues (Δ*E*) remain in the range 0 < Δ*E* < 1, it can be inferred that the hue deviations are invisible to the human eye [75]. It can therefore be established that the modification did not change color of the polymer. Following the analysis of the black recyclate, it can be emphasized that any changes and effects obtained as a result of those modifications may not be unambiguously generalized for recyclates of a different color. The obtained results are applicable exclusively to the black recyclate tested, and it cannot be unambiguously concluded what modification effects would occur for recyclates characterized by other colors.

Gloss is a characteristic of plastic products associated with their surface properties, which defines their decorative and functional properties. Gloss is most often described as an optical sensation which occurs as a result of light reflection and dissipation on the material surface or immediately above it [76,77]. The magnitude of the measured gloss depends on the type of lighting and its absorption, color transparency, refractive index and the type of product surface [77]. For polymeric materials, the amount of reflected light increases with the increase in the angle at which the light strikes the concerned surface, whereas the light which is not reflected penetrates into the material and, depending on its color, undergoes either absorption or dissipated reflection. The variations in gloss magnitudes summarized in Table 3 imply the direct effect of PDMS addition on the parameter under examination. Samples of recyclate with the addition of the modifier were characterized by higher GU values compared to the samples of unmodified polyethylene, which were characterized by a GU value of 59.9 ± 1.06. The mixture containing 2 wt.% PDMS exhibited the highest gloss, which increased its magnitude to as high as 7.3 GU, compared to the unmodified LLDPE. Higher GU magnitudes also occurred for other samples with the added modifier. Considering the standard deviation value, it can be stated that the addition of PDMS in the amount of 0.1, 0.2 and 0.4 wt.%, respectively, has a similar effect on the polyethylene surface gloss. The average gloss for the aforementioned PDMS concentrations improved by approx. 2.6 GU. The measurements have shown that PDMS can modify the gloss of low-density polyethylene recyclate, causing its improvement. It should be borne in mind, however, that the improvement in recyclate gloss may result from the specific surface properties of PDMS, but it can also occur due to the rheological properties of the produced mixtures. The latter scenario involves changes in flows as the mold’s cavity is filled and, in particular, interactions at the polymer–mold surface interface occurring during the process [78].

### 3.3. Mechanical Properties

Among the many requirements imposed on plastic products, the specifications regarding their mechanical properties are a priority. It is necessary to ensure the assumed mechanical characteristics of polymeric products and maintain them on the appropriate level in order to achieve the desired comfort of use and the overriding goal at the design stage. The reasons given above justify the driving force behind this study, namely the need to determine how the modification of polyethylene recyclate with PDMS will influence its mechanical characteristics. To this end, an evaluation of the mechanical parameters of the tests materials under static tension conditions, as well as their impact resistance, was carried out. The results of the measurements of the module of elasticity at tension (*E_f_*), tensile strength (*σ_M_*), elongation at rupture (*ε_B_*) and the Charpy impact resistance (*a_cN_*) carried out for polyethylene recyclate and polyethylene recyclate modified with PDMS (0.1 PDMS–2.0 PDMS), respectively, are summarized graphically in Figure 5. The values of the mechanical parameters of the recyclate and all the test materials are contained in ranges characteristic for pristine LLDPE [13]. In the evaluation of the mechanical properties, it is not possible to show variations in the quantities under discussion, resulting from the LLDPE recycling process, since the material is a commercial recyclate. It can only be inferred that the recyclate used in the tests is a product characterized by satisfactory homogenization and quality level, as the obtained results are distinguished by good repeatability (a low measuring error). A one-way analysis of variance (ANOVA) followed by a Tukey HSD post-hoc test (statistically significant difference at *p* < 0.05) were conducted to compare the mechanical properties among the different samples and to evaluate the significance of the differences between them.

**Figure 8:**
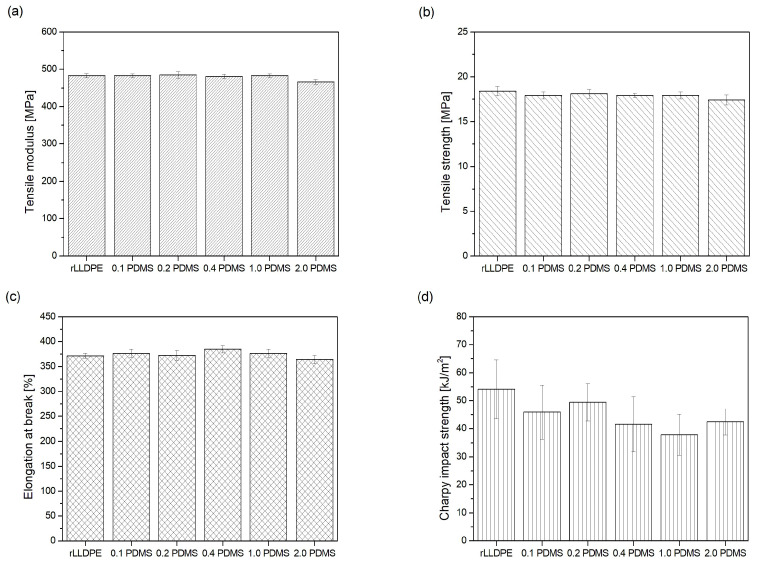
Mechanical properties of rLLDPE and polymer blends containing different concentrations of PDMS (0.1, 0.2, 0.4, 1.0 and 2.0 wt.%): (**a**) tensile modulus; (**b**) tensile strength; (**c**) elongation at break; (**d**) Charpy impact strength.

No significant changes in the concerned mechanical parameters occurred during static tension tests within the adopted ranges of modifier concentrations. In terms of the module of elasticity, its average magnitude remained at a level of approx. 483 MPa for all tested materials (for rLLDPE 483 ± 5.42 MPa), except for the mixture containing 2.0 wt.% PDMS (Figure 8a). For this mixture, a slight decrease in the *E_f_* value down to a level of approx. 466 MPa (*∆E_f_* = 17 MPa) was observed. The statistical analysis indicates that this material is significantly different from all other materials, with *p* < 0.01 for all pairwise comparisons. A similar situation applies to the tensile strength (Figure 8b); in this case, the mean *σ_M_* value for rLLDPE and PDMS-containing mixtures fluctuates at approx. 17.95 MPa. The lowest strength is exhibited by the material containing 2 wt.% of the modifier, amounting to 17.42 MPa. In this case, the statistical analysis revealed that the sample with 2 wt.% of the modifier (2.0 PDMS) was significantly different only from the unmodified sample (*p* < 0.04). However, when considering the value of the standard deviation associated with these measurements (±0.56 MPa), the strength of 2.0 PDMS becomes equal to that of the other mixes under consideration. Additionally, no significant changes in the magnitudes of elongation at break or any visible trends in *ε_B_* variation occurred as a function of modifier content (Figure 8c). The average value of elongation for rLLDPE and its modified mixes amounted to ~375%, and the obtained magnitudes of elongation, after considering the standard deviation, are identical (for rLLDPE *ε_B_* = 371.65 ± 5.29; for 2.0PDMS *ε_B_* = 364.36 ± 8.34). No significant difference was observed in that case. According to the investigation results reported by the authors of the publication [20], the mechanical parameters of high-density polyethylene (HDPE) modified with the addition of PDMS decreased with increasing PDMS content in the adopted modifier concentration range, i.e., from 2 to 20 wt.%. It can be inferred that the reduction in the mechanical parameters of the polyethylene as a result of modifier addition is associated with special interactions between PE–PDMS macroparticles, similar to those occurring in the case of the addition of plasticizers or other slip agents [14,63]—as mentioned during the discussion of the rheological test results. Similar to a plasticizer, the macroparticles of PDMS penetrate into polyethylene chains and reduce the forces of inter- and intramolecular interactions between them, thus lowering their strength. By analyzing the investigation results presented herein and relating them to the publication referred to above [20], it can be concluded that the addition of 2 wt.% of the modifier may represent its limiting concentration, since further increase in the PDMS content beyond this threshold may influence the mechanical properties determined in tensile testing. In the presented investigation, a reduction in the magnitude of the elasticity modulus and tensile strength were observed at the above-mentioned concentration (2 wt.%). This can also be caused by the presence of clusters of PDMS in the structure of the 2.0PDMS blend (see EDS map; Figure 5), which act as notches and reduce the strength of rLLDPE under static tensile conditions. The addition of poly(dimethylsiloxane) changed the impact strength of polyethylene. A decreasing trend in the *a_cN_* value is observed with increasing PDMS concentration in the rLLDPE matrix. In the case of impact strength, the statistical analysis revealed a statistically significant difference between the unmodified sample and samples containing 0.4, 1.0 and 2.0 wt.% PDMS, as well as between the samples containing 0.2 wt.% and 1.0 wt.% PDMS. The impact strength of unmodified polyethylene was equal to 54.09 ± 10.48 kJ/m^2^, while the addition of a 2 wt.% modifier lowered the a_cN_ value to 42.45 ± 4.67 kJ/m^2^. The reduction in impact resistance already occurs for the lowest concentration of the modifier used, i.e., with the addition of 0.1 wt.% (a_cN_ = 45.97 ± 9.63 kJ/m^2^). The above research results indicate that when PDMS is used as a modifier of the surface properties of rLLDPE, its unfavorable effect on the impact strength of the polymer should be taken into account, even at low concentrations. Therefore, it can be stated that, the addition of PDMS within the considered concentration range does not significantly impair the quality of the recyclate from the perspective of its mechanical parameters under static tensile conditions. Instead, it affects the deterioration of its impact resistance.

## 4. Conclusions

A prerequisite for increasing the use of recycled polymer materials in plastics processing is to make them more attractive, relative to the original raw materials. One of the methods for improving the functional properties of recyclates, in addition to techniques aimed at improving their purity, is their modification. Within the framework of the research results presented in this article, it was shown that PDMS can be an effective modifier of the surface properties of linear low-density polyethylene recyclate derived from post-production waste in the rotational molding process, even at low concentrations. The activity of the modifier, which causes an increase in the hydrophobic character of the recyclate surface (an increase in the value of the water wetting angle) and a decrease in the dynamic coefficient of friction, was manifested over the entire range of the tested concentrations. The enhancement of the above-mentioned properties can also improve the economics of processing the modified recyclate in the rotational molding process, resulting from a reduction in the processing time due to the easier removal of the product from the mold. The addition of PDMS also allowed for improvement in one of the most important parameters determining the surface aesthetics of plastic products, namely gloss. This study also showed a slight effect of the modification on reducing the hardness of the rLLDPE surface, both the hardness determined by point (Shore hardness) and the scratch hardness. In the case of scratch hardness, it was shown that the nature of the resulting damage (scratches) varied, depending on the amount of modifier used. An increase in its concentration caused an increase in the width of the scratch, while reducing the plastic deformation in its structure, which contributes to improved sliding properties of the modified polymer. Additionally, PDMS used as a modifier positively affects the reduction in water absorption of polyethylene recyclate, which contributes to a decrease in its drying time before processing, and possibility enables its complete elimination when the highest concentrations of PDMS are used. No effect of the modifier addition on the mass flow rate of the recyclate, i.e., its processability, was observed in this study. However, the use of a multipoint measurement technique (rotational rheometer) allowed the demonstration of a slight effect of the carried-out modification on the viscosity of polyethylene, shifting it towards lower values as the PDMS content in the mixture increased. This modifier activity can contribute to a potential improvement in the surface quality of rotationally molded products, reducing the ‘pin-holes’ effect. An unfavorable effect of the addition of PDMS in the area of processing properties was noted in the case of processing shrinkage, which should be taken into account during tool design. A very important observation, which highlights the rather selective nature of the modifier used, is its lack of effect in terms of the tensile parameters of rLLDPE under static tensile conditions. Summarizing the results of the tests conducted, it can be clearly stated that PDMS has confirmed its effectiveness as a modifier of surface properties within the adopted concentration range, also in the case of recycled materials. The effectiveness of its action was evident even at low concentrations and contributed to improvement in some of the processing properties of the polymer, which may be an additional aspect that justifies the use of PDMS in rotational molding processes based on recycled raw materials.

## Figures and Tables

**Table 1:** The composition of investigated materials.

Symbol	Content in wt. %		
	rLLDPE	LDPE	PDMS
LDPE/PDMS	0	50	50
rLLDPE	100	0	0
0.1 PDMS	99.8	0.1	0.1
0.2 PDMS	99.6	0.2	0.2
0.4 PDMS	99.2	0.4	0.4
1.0 PDMS	98.0	1.0	1.0
2.0 PDMS	96.0	2.0	2.0

**Table 2:** The processing properties of investigated materials.

Polymer Materials	Moisture Content [wt.%]	*MFR* [g/10 min]	Zero Shear Viscosity [Pa·s]	Longitudinal Processing Shrinkage [%]	
				16 h	3 Month
rLLDPE	0.130 ± 0.0119	4.04 ± 0.033	3010	2.11 ± 0.117	2.22 ± 0.105
0.1 PDMS	0.122 ± 0.0091	3.93 ± 0.066	2930	2.19 ± 0.129	2.37 ± 0.068
0.2 PDMS	0.086 ± 0.0274	4.06 ± 0.036	2840	2.35 ± 0.103	2.44 ± 0.102
0.4 PDMS	0.085 ± 0.0280	3.94 ± 0.022	2830	2.39 ± 0.052	2.50 ± 0.083
1.0 PDMS	0.045 ± 0.0188	3.92 ± 0.049	2780	2.43 ± 0.066	2.54 ± 0.075
2.0 PDMS	0.017 ± 0.0065	3.98 ± 0.036	2640	2.51 ± 0.177	2.61 ± 0.180

**Table 3:** The surface properties of investigated materials.

Polymer Materials	Wetting Angle [°]	Dynamic Coefficient of Friction [/]	Shore D Hardness [Sh°]	Scratch Hardness [MPa]	Gloss [GU]
rLLDPE	88.25 ± 0.257	0.13 ± 0.011	50.0 ± 0.27	79.8 ± 0.39	59.9 ± 1.06
0.1 PDMS	90.27 ± 0.767	0.12 ± 0.006	49.7 ± 0.45	63.2 ± 0.24	62.1 ± 1.72
0.2 PDMS	91.14 ± 0.681	0.10 ± 0.009	50.0 ± 0.03	61.9 ± 0.19	62.3 ± 1.29
0.4 PDMS	94.96 ± 0.640	0.09 ± 0.006	50.0 ± 0.00	61.3 ± 0.12	63.0 ± 1.82
1.0 PDMS	96.94 ± 0.693	0.08 ± 0.008	49.1 ± 0.09	58.2 ± 0.09	65.9 ± 1.38
2.0 PDMS	102.47 ± 0.753	0.06 ± 0.004	48.0 ± 0.27	55.7 ± 0.20	67.2 ± 1.01

## Data Availability

The original contributions presented in this study are included in the article. Further inquiries can be directed to the corresponding authors.

## Abbreviations

The following abbreviations are used in this manuscript:

HDPE	high-density polyethylene
LDPE	low-density polyethylene
LLDPE	linear low-density polyethylene
*MFR*	melt flow rate
PDMS	polydimethylsiloxane
PE	polyethylene
rLLDPE	recyclate of high-density polyethylene
*S_L_*	longitudinal processing shrinkage
UHMW	ultra-high molecular weight
